# Redox signaling and Alzheimer’s disease: from pathomechanism insights to biomarker discovery and therapy strategy

**DOI:** 10.1186/s40364-020-00218-z

**Published:** 2020-09-11

**Authors:** Yuan-Yuan Chen, Min-Chang Wang, Yan-Ni Wang, He-He Hu, Qing-Quan Liu, Hai-Jing Liu, Ying-Yong Zhao

**Affiliations:** 1 Faculty of Life Science & Medicine, Northwest University, No. 229 Taibai North Road, Xi’an, 710069 Shaanxi China; 2 Instrumental Analysis Center, Xi’an Modern Chemistry Institute, Xi’an, 710065 Shaanxi China; 3 Beijing Hospital of Traditional Chinese Medicine, Capital Medical University, Beijing, 100010 China; 4 Shaanxi Institute for Food and Drug Control, Xi’an, 710065 Shaanxi China

**Keywords:** Alzheimer’s disease, Inflammation, Oxidative stress, Neurodegenerative disease, Metabolomics

## Abstract

**Abstract:**

Aging and average life expectancy have been increasing at a rapid rate, while there is an exponential risk to suffer from brain-related frailties and neurodegenerative diseases as the population ages. Alzheimer’s disease (AD) is the most common neurodegenerative disease worldwide with a projected expectation to blossom into the major challenge in elders and the cases are forecasted to increase about 3-fold in the next 40 years. Considering the etiological factors of AD are too complex to be completely understood, there is almost no effective cure to date, suggesting deeper pathomechanism insights are urgently needed. Metabolites are able to reflect the dynamic processes that are in progress or have happened, and metabolomic may therefore provide a more cost-effective and productive route to disease intervention, especially in the arena for pathomechanism exploration and new biomarker identification. In this review, we primarily focused on how redox signaling was involved in AD-related pathologies and the association between redox signaling and altered metabolic pathways. Moreover, we also expatiated the main redox signaling-associated mechanisms and their cross-talk that may be amenable to mechanism-based therapies. Five natural products with promising efficacy on AD inhibition and the benefit of AD intervention on its complications were highlighted as well.

**Graphical Abstract:**

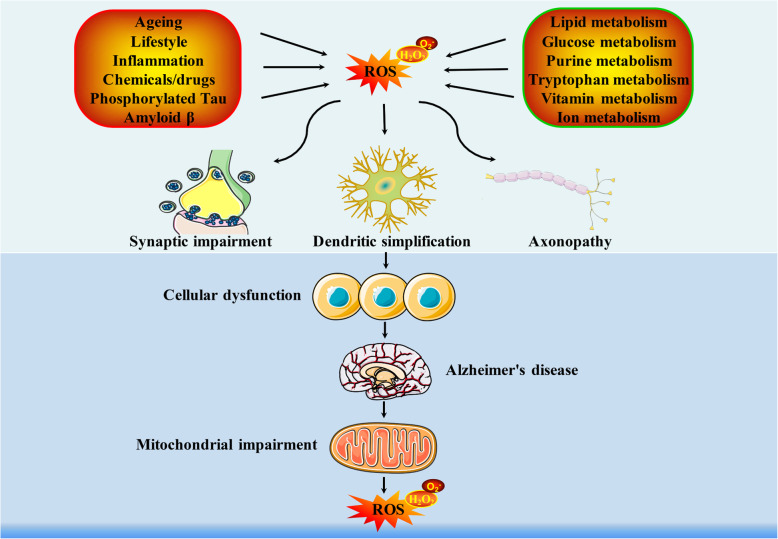

## Highlights

- Alzheimer’s disease is a disease with expectation to be major challenge in elders.
- No clinical trial in 1984–2017 has provided any improvement in disease progression.
- ROS is implicated in pathogenesis and may contribute to mechanism-based therapies.
- Five natural products with huge potential in halting disease are also highlighted.
- Alzheimer’s disease intervention is instrumental to other disease recovery as well.

## Introduction

**Fig. 1:**
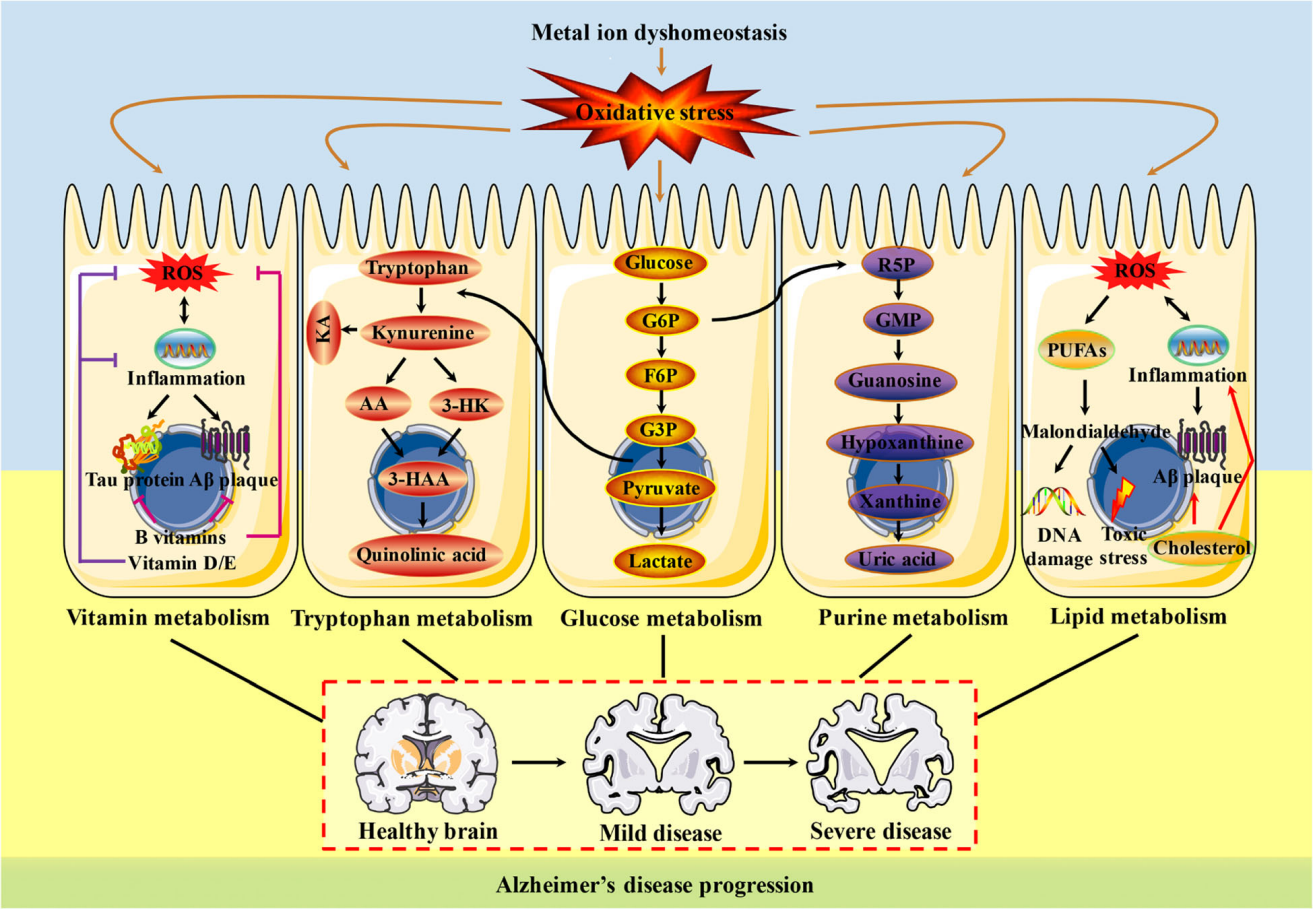
The primary oxidative stress-associated metabolic pathways in AD. AD is a neurodegenerative disease with a projected expectation to blossom into the major challenge in aging populations, while there is a severe paucity of both diagnosis and treatment for this disease. A series of metabolites with relevance to oxidative stress, including glucose metabolism, lipid metabolism, purine metabolism, tryptophan metabolism, vitamin metabolism and metal ion metabolism, have been major risk factors for AD progression, the intervention of which may provide novel insight into the development of mechanism-based therapies. AA: Anthranilic acid; F6P: fructose 6-phosphate; G3P: glyceraldehyde 3-phosphate; G6P: glucose 6-phosphate; GMP: guanosine-5′-monophosphate; 3-HAA: 3-hydroxyanthranilic acid; 3-HK: 3-hydroxykynurenine; KA: Kynurenic acid; PUFAs: polyunsaturated fatty acids; R5P: ribulose 5-phosphate

Alzheimer’s disease (AD) is a complex/chronic aging related neurodegenerative disorder that causes cognitive defects and gradual memory loss [1]. The global burden of neurological diseases has increased substantially during 1990–2015 due to ageing and expanding population numbers [2] and all clinical trials between 1984 and 2017 have failed to provide any improvement in clinical outcomes [3], suggesting mechanism-based therapies are pressingly needed. Hyperphosphorylated tau protein [4], β-amyloid (Aβ) aggregation [5], aging [6], and importantly, inflammation [7] and oxidative stress [8] are tightly implicated in AD neurodegeneration. Cellular stresses or normal metabolic processes continuously generate reactive oxygen species (ROS) and a basal level of oxidative stress is of great significance to cell survival, while severe oxidative stress inevitably results in widespread oxidative damage [9]. Neuronal cells are metabolically active cells that utilize almost one fourth of total oxygen in the body, which are particularly susceptible to free radical attack induced degenerations [10]. A weakening in anti-oxidant defense systems and increased ROS generation are most common in elders, indicating the elders are most affected by redox signaling associated degenerative diseases. Metabolites can offer possible avenues for disease prevention and treatment since they are significant risk factors for AD progression both in terms of changes in metabolism and metabolic deficiencies, which has been promising in the field of inspiring drug discovery [11, 12]. Here, we primarily outline the current knowledge regarding the relationship between redox signaling and metabolic pathways in AD, aiding the discovery of potential targets and the development of mechanism-based therapies (Fig. 1).

## The potential association between redox signaling and metabolic pathways

### Lipid metabolism

Inflammation and oxidative stress are interrelated factors deeply implicated in the pathogenesis of AD, and excessive ROS inevitably lead to lipid damage [13]. Polyunsaturated fatty acids are rich in the brain [14], and they are particularly prone to peroxidization owing to their high reducibility. ROS could degrade polyunsaturated fatty acids into malondialdehyde [15], which causes DNA damage and toxic stress in cells [16]. Moreover, the balanced levels of inflammation and oxidative stress are favored in lipid accumulation since excessive ROS enhance lipid peroxidation whereas their low levels promote lipid biosynthesis [17]. Snowden et al. showed that five unsaturated fatty acids, including linolenic acid, linoleic acid, eicosapentaenoic acid, arachidonic acid and oleic acid were significantly reduced in the inferior temporal and middle frontal gyri of AD patients compared to healthy controls, while docosahexaenoic acid was significantly increased, which may aid in AD diagnosis [18]. Furthermore, polyunsaturated fatty acids could participate in myriad signal transduction within the brain directly or after enzymatic conversion to a series of mediators, and future studies aimed at clarificating how polyunsaturated fatty acids is altered in brain disorders and developing methods to restore polyunsaturated fatty acid metabolism might portend novel paradigm in AD prevention and treatment [19].

Cholesterol homeostasis was impaired in AD as well [20]. One of the primary risk factors for AD is the presence of apolipoprotein E (APOE), a polymorphic lipoprotein that mainly carries cholesterol in the brain [21]. There are three major APOE alleles in humans, among which APOE_2_ allele is closely associated with the reduced risk of AD, while APOE_4_ allele devotes much to AD occurrence [22]. In addition, cholesterol is of paramount importance to the γ-secretase cleavage of amyloid precursor protein, the last step of Aβ formation [23], and cholesterol depletion could lessen AD by inhibiting Aβ generation [24], providing new insight into AD regression. Cholesterol also contributed to AD pathogenesis by inducing interleukin 1 β production through cytoplasmic sensor NLRP3, while CD36 inhibition ameliorated AD by alleviating inflammation and protecting from the toxic effects of Aβ [25], hinting CD36 intervention may provide additional benefits to disease control and drug development.

Except for mentioned-above factors, Mapstone et al. identified a set of ten blood-based metabolites including PCs (PC diacyl (aa) C36:6, PC aa C38:6, PC aa C38:0, PC aa C40:6, PC aa C40:2, PC aa C40:1 and PC acyl-alkyl (ae) C40:6), acylcarnitines (ACs) (C16:1-OH and Propionyl AC (C3)) and lysophophatidylcholine (lysoPC a C18:2) that predicted phenoconversion to either AD or amnestic mild cognitive impairment in older adults (age ≥ 70) within a 2–3 year with over 90% accuracy [26]. Notably, this is the first time that a plasma biomarker panel with very high accuracy in detecting preclinical AD has been published. It is of paramount importance to AD diagnosis since there are almost no disease-modifying therapies or cures partly due to the inability to detect AD before it progressed to evident functional decline and memory loss.

### Glucose metabolism

Mitochondrial plays a prominent role in ROS generation and the process most affected by ROS overproduction is glycolysis [27]. Liguori et al. discovered that the cerebrospinal fluid (CSF) lactate concentration of 145 patients with AD was increased compared to 80 healthy controls, while it seemed to decrease in parallel with cognitive dysfunction since CSF lactate concentration was higher in 67 patients with mild than that of 78 moderate–severe AD patients, highlighting the clinical potential of CSF lactate concentration as a simple and helpful tool to better define the damage of neuronal brain metabolism in patients with AD [28]. In addition, there is a significant relationship between cerebral glucose hypometabolism and elevated CSF lactate in brain areas typically showing AD neurodegeneration, suggesting neural glucose hypometabolism might affect the cognitive efficacy by damaging brain energetic machine [29]. Moreover, fructose-1,6-bisphosphate is another glycolytic intermediate that shows neuroprotective effect against various harmful conditions in many brain injury models [30]. Particularly, fructose-1,6-bisphosphate could improve cerebral metabolic outcomes via ameliorating inflammation and oxidative stress and preserving glucose metabolism integrity in sepsis [31]. Unfortunately, the effect of fructose-1,6-bisphosphate on AD is rarely covered.

Tricarboxylic acid cycle was also altered in AD cases [32]. High CSF pyruvate has been widely reported in AD patients compared with healthy elderly controls [33], while plasma pyruvate was significantly reduced in Alzheimer’s-like mice [34]. Furthermore, chronic treatment with pyruvate could alleviate short and long-term memory deficits via other pathogenic pathways without reducing amyloid- and tau-dependent pathology in preclinical AD models, and pyruvate thus has the potential to be exploited as an alternative therapeutic agent that cooperates with drugs directly address amyloid- and tau-dependent mechanisms [35].

### Purine metabolism

Guanosine is a purine nucleoside that shows neuroprotective effects on preventing aging-related diseases by modulating cellular redox status/glutamatergic system [36]. Tasca et al. discovered that guanosine could attenuate Aβ-induced neuroinflammation/oxidative stress in a series of in vitro models [37], suggesting guanine may be a promising compound with neuroprotective properties in AD treatment. Guanosine were implicated in AD stratification as well. Alonso-Andres et al. discovered that guanosine was significantly reduced in frontal cortex at early stages (AD I–II) and remarkably increased in parietal cortex at advanced stages (AD V–VI), while it was significantly increased in temporal cortex at both early stages (AD I–II) and advanced stages (V–VI), which may be exploited for AD diagnosis [38].

Hypoxanthine is another purine compound that implicated in AD progression. Acetylcholinesterase is closely associated with AD pathophysiology and hypoxanthine could enhance acetylcholinesterase activity when it is added to incubation medium [39]. Constant stimulation of acetylcholinesterase activity might reduce acetylcholine levels, an essential neurotransmitter of central nervous system, and hypoxanthine therefore contributes much to memory deficits by acetylcholinesterase-related mechanism. Hypoxanthine also promotes AD development via inducing inflammation and oxidative stress, hinting patients with high hypoxanthine is susceptible to AD [40]. Li et al. discovered hypoxanthine was significantly elevated in the brain of AD mice [41], which may be helpful for AD discrimination. However, Alonso-Andres et al. found hypoxanthine was significantly reduced in frontal cortex at early stages (AD I–II), which is quite diffetent from previous studies [38]. Considering the fact that determinations in the brain do not reflect events in particular brain region and available animal models cannot absolutely recapitulate relevant human diseases, the alteration of hypoxanthine in AD remains to be further valited.

Uric acid is the end-product of purine metabolism that inversely associated with the risk of AD based on its anti-oxidative property, suggesting the neuroprotective role of uric acid on disease progression [42]. Lu et al. uncovered that individuals with a medical history of gout had a 24% lower risk of AD after adjustment for sex, age, BMI, lifestyle factors, socioeconomic status, prior cardiovascular-metabolic conditions and the use of cardiovascular drugs for over 5 years follow up, providing the first population-based evidence that gout was inversely associated with AD risk and supporting the potential protective role of uric acid [43]. Nevertheless, the relationship between uric acid and AD remains debated since Augustin et al. found the risk of dementia, especially for mixed or vascular dementia, might be increased with high uric acid in a population-based cohort study for over 12 years follow up [44], indicating the controversial role of uric acid on AD cannot yet be dismissed.

### Tryptophan metabolism

Neuroactive metabolites in kynurenine pathway via tryptophan metabolism have been shown great association with neurodegenerative disorders, the hyperfunction or hypofunction of which made enormous contributions to AD progression and effective interventions may be therapeutically benificial to disease recovery [45]. Four metabolites that closely associated with AD were described in detail.

3-hydroxykynurenine is a neurotoxic metabolite that plays a critical role in neurocognitive impairments [46], the supression of which may expand our armaments to win more battles against AD. Kynurenine 3-monooxygenase contributed much to 3-hydroxykynurenine generation and the efficacy of kynurenine 3-monooxygenase inhibitor arised from normalizing the imbalance of neurotoxic and neuroprotective metabolites [47]. Indeed, 2-(3,4-dimethoxy benzenesulfonylamino)-4-(3-nitrophenyl)-5-(piperidin-1-yl)methylthiazole, a bioavailable prodrug of Ro 61–8048 (the most widely used kynurenine 3-monooxygenase inhibitor with unstable metabolic stability), selectively restrained peripheral kynurenine 3-monooxygenase and ameliorated neurodegeneration in well-established mouse model of AD via modestly elevating neuroprotective metabolite without increasing neurotoxic metabolites [48]. Therefore, it could avoid potential adverse effects and may be a more safer and attractive therapeutic agent. Nevertheless, Beconi et al. discovered that Ro-61-8048 concentrations were similar after 0.05 mg/kg Ro-61-8048 alone or coadministered with 10 mg/kg 2-(3,4-dimethoxy benzenesulfonylamino)-4-(3-nitrophenyl)-5-(piperidin-1-yl)methylthiazole in mice, hinting it was not a prodrug for Ro-61-8048 and its efficency on neurodegeneration by inhibiting kynurenine 3-monooxygenase remains to be determined by far [49].

Quinolinic acid, another endogenous neurotoxin, was increased in the serum and CSF of AD patients, and the most significant factor responsible for quinolinic acid elevation was aging, suggesting kynurenine pathway is activated during aging by modulating neuroinflammation as aging is associated with inflammatory phenotype and inflammation acts a critical activator of kynurenine pathway [50]. In addition, N-methyl-D-aspartate receptor (NMDAR) is a glutamate receptor that has neurotoxicity and neurotrophic effects, both the hypofunction and excitotoxicity of which are implicated in neurodegeneration [51]. Quinolinic acid could deteriorate AD by activating NMDAR, accelerating ROS generation and promoting hyperphosphorylated tau proteins formation [52], suggesting quinolinic acid inhibition may provide additional benefits to AD treatment. D-amino acid oxidase (DAO) also plays a pivotal role in AD by regulating NMDAR and one of the important avenues to elevate NMDAR activity is via inhibiting DAO [53]. Sodium benzoate, a prominent DAO inhibitor, significantly ameliorated the cognitive impairment of patients with early-phase AD without evident side-effects [53]. Sodium benzoate has also been reported to have anti-oxidant effects by inhibiting ROS production [54] and increasing the activity of catalase [55]. Moreover, pLG72 or DAO activator was increased in patients with early-phase dementia with the highest level at mild AD, while it was decreased with the severity of cognitive decline in later-phase AD, indirectly supporting the hypothesis of hypo-NMDAR in early-phase AD and glutamate excitotoxicity in late-phase AD [56]. Furthermore, Lin et al. found the level of peripheral DAO might increase with age-related cognitive deficit for the first time [57], and the alteration of plasma D-amino acids including D-aspartate was closely associated with AD progression [58], which may be exploited as potential biomakers for AD. Considering AD is a complex disease and collecting peripheral blood is more feasible than samples from brain tissues or CSF, it may be favorable to combine DAO, DAO activator and D-amino acids for assisting the diagnosis.

Kynurenic acid is a neuroprotective metabolite in kynurenine pathway. Emerging evidence revealed that kynurenic acid was reduced in the CSF of 33 AD patients compared to 39 age-matched controls [50] and neurodegeneration was markedly ameliorated by shifting kynurenine pathway flux toward kynurenic acid synthesis [59]. However, van et al. uncovered that kynurenic acid was significantly increased in the CSF of 40 AD patients compared to 34 healthy controls [60], which is not consistent with previous studies describing reduced kynurenic acid levels in CSF of AD patients. These discrepancies may be explained by age and gender differences between patients and control subjects since age was slightly unbalanced in the latter and they did not take better account of gender. Kynurenic acid could restrain the excitotoxicity of NMDAR as well. This is very valuable for pharmaceutical exploitation since NMDAR antagonist approved for clinical use is limited and myriad compounds concerning NMDAR inhibition have been explored in vain [61]. Besides its anti-excitotoxicity, kynurenic acid also has anti-inflammatory and anti-oxidant effects via reducing pro-inflammatory cytokines release and improving ROS scavenging [61]. Of note, although kynurenic acid has been shown to possess neuroprotective effect, its role in neurogenic progenitors remains unclear. Christos et al. found kynurenic acid impaired neural stem cell plasticity [62]. This finding is very important because current clinical effort for enhancing kynurenic acid levels may be helpful for neuronal survival but suppress the neurogenic outcome, which can help to propose refinements on drug administration and clinic practice. Collectively, kynurenic acid is a potential multitarget to normalize the disturbed kynurenine pathway and thus alleviate AD pathogeneses, while its application remains challenging.

Anthranilic acid is an endogenous redox active metabolite that retards cognitive disfunction due to its ability on Fe coordination complex formation and ROS scavenging, and anthranilic acid modulation therefore represents a promising therapeutic approach in AD intervention [63]. Anthranilic acid also exhibits anti-inflammatory effect either by itself or its 5-hydroxylated metabolites [61]. An expected anti-inflammatory property of anthranilic acid is rooted in the fact that anthranilic acid is a precursor of some anti-inflammatory drugs as exemplified by mefenamic acid [64]. Additionally, Kwon et al. revealed that oscarellin, a new anthranilic acid derivative isolated from a Philippine sponge, could diminish pro-inflammatory cytokines such as extracellular ERK1/2 and nuclear factor-κB (NF-κB), while anti-inflammatory cytokine of transcription factor-3 was enhanced, indicating anthranilic acid may also attenuate inflammation via modulating inflammatory cytokines [65]. As such, considering the prominent role of inflammation and oxidative stress in cognitive dysfunction, anthranilic acid might be a potential therapeutic target to fight against AD.

### Vitamin metabolism

**Fig. 2:**
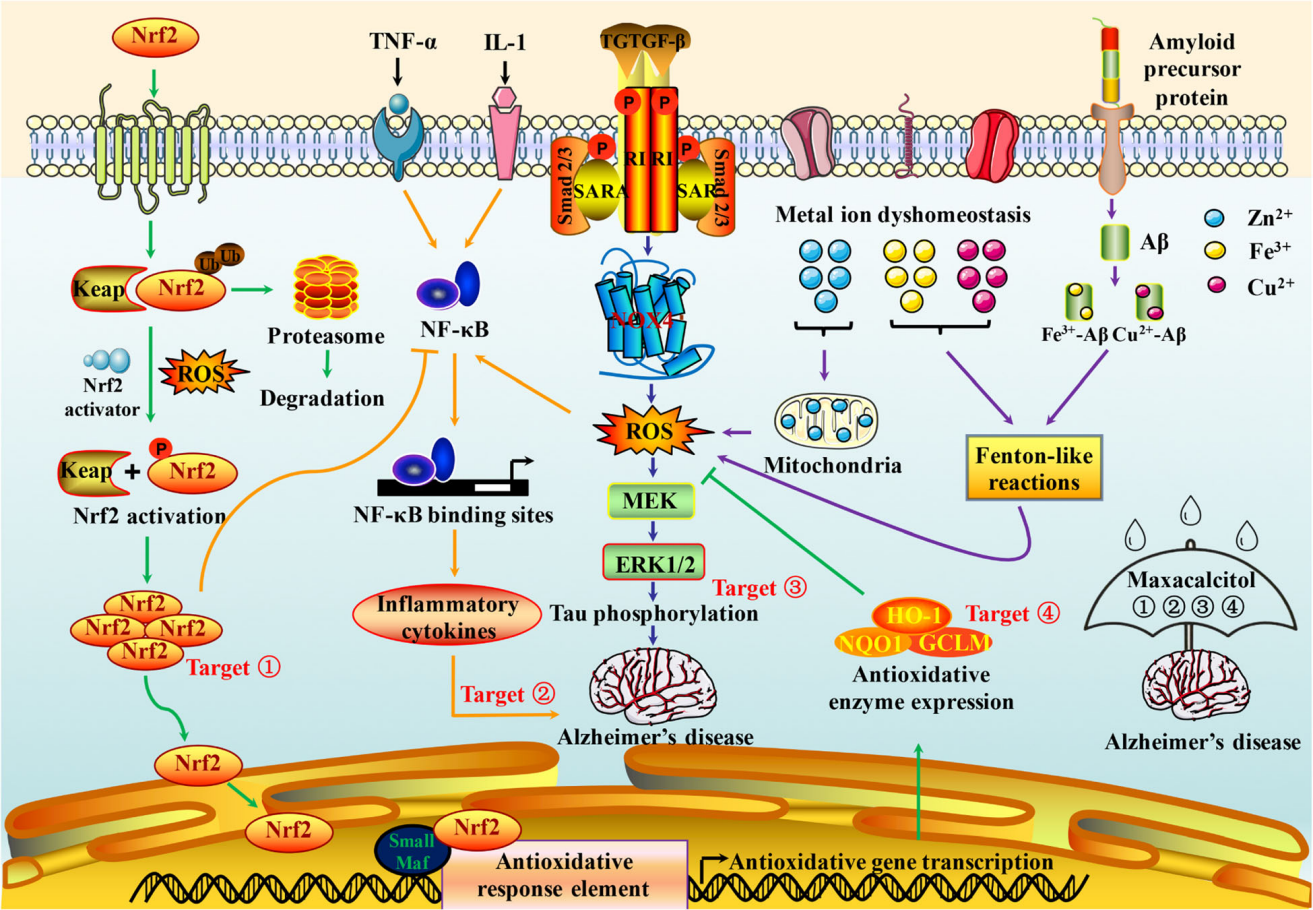
The main redox signaling-associated mechanisms and their cross-talk in AD progression. NOX, TGF-β, NF-κB and Nrf2 are remarkable mediators of oxidative stress that implicated in AD development. NOX is dedicated contributor Aβ-induced ROS generation, and NOX signaling pathway is closely associated with Aβ deposition and cognitive deficits. TGF-β/Smad signaling also promotes ROS production, and NOX4 is the main cause of TGF-β induced ROS generation via TGF-β/Smad/ROS signaling cascade. In addition, tau protein hyperphosphorylation is another hallmark of AD, which could deteriorate AD through TGF-β/Smad/NOX4/ERK1/2/tau protein cascade. Moreover, metal ions and NF–κB also contributes to AD progression by accelerating ROS and inflammation respectively, while Nrf2 shows potential protective effect against AD via promoting anti-oxidant responses and inhibiting NF–κB. Maxacalcitol is a vitamin D analogue that significantly alleviates cognitive impairment of AD rats through elevating Nrf2, restraining inflammation and reducing the hyperphosphorylation of tau proteins. ERK: extracellular signal-related kinase; GCLM: glutamate-cysteine ligase modifier subunit; HO-1: haem oxygenase-1; Keap1: Kelch-like ECH-associted protein 1; MEK: mitogen-activated protein kinase/extracellular signal-related kinase; NQO1: NAD(P)H dehydrogenase quinone 1, SARA: smad anchor for receptor activation

The enzyme responsible for vitamin D generation and vitamin D receptor are widespread in human brain especially for areas related to neuropsychological function, and vitamin D may therefore play a significant role in neurodegenerative diseases. The plasma 25-hydroxyvitamin D of AD patients seemed to be markedly lower compared to healthy controls of the same age [66], while daily oral 800 IU vitamin D for 12 months ameliorated AD and decreased Aβ-related biomarkers in randomised, double-blind, placebo-controlled trial [67], suggesting vitamin D has potential benefits on cognitive recovery. Vitamin D is implicated in AD onset as well. Littlejohns et al. discovered that there was strong association between the risk of AD and baseline vitamin D concentrations through both vascular and neurodegenerative mechanisms over a mean of 5.6 years follow up, whereas the optimal vitamin D level for general health remains to be determined [68]. The neuroprotective benefit of vitamin D was likely attributed to its anti-oxidant/anti-inflammatory effects [69] and exercise could enhance the efficacy of vitamin D therapy [70]. El-Din et al. demonstrated that Nrf2 and its downstream anti-oxidant effectors were decreased in AD rats, while the neuro-inflammation as evidenced by TNF-α and phosphorylated ERK1/2 that led to the hyperphosphorylation of tau protein were increased (Fig. 2) [71]. Maxacalcitol, a vitamin D analogue, significantly improved cognitive impairment of AD rats via elevating Nrf2 signaling pathway as well as reducing the hyperphosphorylation of ERK1/2 and tau proteins [71], making AD more curable than inevitable (Fig. 2).

Plasma homocysteine concentration increased with age in normal human and an elevated plasma homocysteine level was an independent, strong risk factor for AD development [72]. Although no clear mechanisms between B vitamin intake and cognitive decline have been well established, a series of biologically plausible mechanisms have been widely proposed to explain the effect of one-carbon metabolism relevant B vitamins, including vitamin B_12_, vitamin B_6_ and folate on cognitive impairment [73]. Deficiencies in any of above-mentioned B vitamins may raise blood concentration of homocysteine via perturbing one-carbon metabolism and causing low enzymatic activities for homocysteine remethylation or trans-sulfuration, which contributes to oxidative damage and subsequent cognitive decline [74]. Hyperhomocysteinemia induced memory deficits with AD-like Aβ and tau pathologies in the hippocampus as well, while folate/vitamin B_12_ supplementation could improve memory by preventing hyperhomocysteinemia induced AD-like pathologies, highlighting B vitamins may be a preventive or even therapeutic alternative against AD [75]. In addition, Guo et al. demonstrated that hyperhomocysteinemia induced Aβ accumulation and tau hyperphosphorylation was found in the retina as well, while simultaneous B vitamins supplementation efficiently reduced plasma AD-like hyperhomocysteinemia with attenuation of AD-like Aβ and tau pathologies in the retina for the first time [76]. Since the accumulation of Aβ and hyperphosphorylated tau is the main lesion of AD in the hippocampus, the efficacy of drugs cannot be visualized at the early stage, and the retina may therefore become an accurate and non-invasive visualization window for the early detection of AD-like pathologies and evaluating the intervention effects of B vitamins on AD. Nonetheless, randomized studies in individuals with normal vitamin levels and mild to moderate AD have yielded conflicting results. High-dose B vitamins supplementation might be useful in older patients with relatively high homocysteine levels, while individuals with normal vitamin levels and mild to moderate AD were unlikely to benefit from B vitamins supplements [77]. Hence, B vitamins treatment are recommended in patients with elevated homocysteine and studies in more narrowly defined AD are warranted.

Moreover, glucose hypometabolism is an invariant neurodegenerative hallmark that has significant diagnostic value in AD. Sang et al. discovered that the reduction of thiamine diphosphate, a critical coenzyme of glucose metabolism, tightly correlated with brain glucose hypometabolism for the first time, providing novel insight into the pathogenesis of AD [78]. Thiamine also acts as a free redical scavenger, and deficient thiamine-dependent motochondrial dehydrogenase complexes accelerate AD by producing oxygen free radicals [79]. Although the reduction in thiamine diphosphate and thiamine diphosphate–dependent enzymes has been widely reported both in autopsied brain and blood samples of AD patients [80–82], the diagnostic value of blood thiamine metabolites has rarely been covered. Pan et al. firstly showed that the alteration of blood thiamine metabolites could serve as a promising biomarker in AD diagnosis with high sensitivity and specificity since thiamine diphosphate levels were significantly reduced in AD patients in both exploration phase and validation phase [83]. Additionally, high performance liquid chromatography fluoroscopy is an ideal tool for AD diagnosis with noninvasive, reliable, inexpensive and simple to perform merits, which is very suitable for studies in large populations.

Vitamin E is a powerful anti-oxidant that protects against free radicals induced AD [84]. Mounting evidence that free radicals had prominent effect on deteriorating neurodegeneration has lead to increasing studies in utilizing vitamin E to help cure patients with AD. Sano et al. firstly showed the effectiveness of vitamin E supplement (2000 IU/d) on disease control in participants with moderately severe AD [85], while it had no benefit in patients with mild cognitive impairment compared with placebo groups [86]. Maurice et al. firstly studied the effect of vitamin E (2000 IU/d) on patients with mild-to-moderate AD and indicated that vitamin E showed significant benefit in retarding cognitive decline compared with the placebo, which could be a good candidate for AD treatment [87]. Although the effect of vitamin E (2000 IU/d) on different periods of AD have been investigated in double-blind, randomized and placebo-controlled clinical trials, it is a great pity that the underlying mechanisms are rarely covered. Wang et al. found α-tocopherol quinine can ameliorate memory deficits by inhibiting pro-inflammatory cytokines as exemplified by interleukin-6 and redox signaling-mediated NF-κB pathway in transgenic AD mice, which may aid our understanding in the efficacy of vitamin E on AD [88]. However, besides the unambiguous beneficial effects of vitamin E, it also accelerated Aβ production and suppressed Aβ degradation with the increase of vitamin E intake, which may help to understand the controversial role of vitamin E supplement in AD treatment [89].

### Metal ion metabolism

Metal ion dyshomeostasis is a well-acknowledged feature of AD [90]. Metal ions (especially for zinc, iron and copper) were found in Aβ aggregation and the concentrations of zinc, iron and copper were significantly increased in brain samples of AD patients compared with age-matched, healthy brain tissues [91]. Aβ accumulation played vital roles in AD pathology and metal ions can coordinate with Aβ, leading to metal-Aβ complexes generation and AD progression [92]. Furthermore, metal ions promoted AD development due to their potential involvement in ROS overproduction. Copper and iron contributed to ROS generation by Fenton-like reactions, while zinc was observed to retard oxidative phosphorylation in mitochondria, causing zinc-triggered ROS production (Fig. 2) [20]. The critical role of redox-active metals in AD pathogenesis strongly argued that amyloid-specific metal be exploited as possible therapeutic targets for this horrible disease [93]. Of note, although metal chelation is recognized as a promising therapy for AD treatment [94], the widespread clinical use of chelators remains a huge challenge as most chelators possess limited efficacy to differentiate toxic metals that tightly associated with Aβ plaques from those required by metal homeostasis [95]. Fortunately, the emergence of a novel electrically controlled-release drug delivery platform that selectively retard metal-induced Aβ aggregation, could effectively inhibit Aβ aggregation, protect cells from Aβ-related cytotoxicity and decrease cellular ROS with no significant cytotoxic effects, which dramatically promoted the design of noninvasive remote-controlled therapeutics for AD treatment [96].

### The main redox signaling-associated mechanisms and their cross-talk in AD

Nicotinamide adenine dinucleotide phosphate oxidase (NOX), transforming growth factor-β (TGF-β), NF-κB and nuclear factor-erythroid 2 related factor 2 (Nrf2) are prominent mediators of oxidative stress [97], which play important roles in regulating AD development. NOX is the primary source of fibrillar Aβ-induced ROS generation, suggesting the elimination of Aβ-induced oxidative damage by inhibiting NOX may provide an attractive therapeutic target for AD treatment [98]. Bruce-Keller et al. discovered that NOX4 was significantly elevated in APP × PS1 transgenic mice in an age-depedent manner, and NOX-associated pathways were intimately associated with both the deposition of Aβ and the loss of cognitive function [99]. TGF-β was implicated in redox signaling as well and NOX4 was most responsible for TGF-β induced ROS generation by TGF-β/Smad/ROS signaling cascade [100]. In addition to its effect on ROS production, TGF-β also induced AD development by initiating or promoting amyloidogenesis [101]. Moreover, tau protein hyperphosphorylation is another hallmark of AD, and phosphorylated ERK1/2 could deteriorate AD through TGF-β/Smad/NOX4/ERK1/2/tau protein cascade. Additionally, NF-κB [102] and metal ions [20] aggravated AD via accelerating inflammatory responses and ROS generation respectively, while Nrf2 protected from AD-like pathological changes via promoting anti-oxidant responses and inhibiting NF-κB activation. The repressor of Nrf2, Kelch-like ECH-associted protein 1, inhibited Nrf2 expression and stimulated its degradation, while Kelch-like ECH-associated protein 1 knockdown increased Nrf2 expression, thereby increasing the anti-oxidant capacity and alleviating Aβ-induced cell damage [103].

## The therapeutic opportunities for natural products in AD and its complications

### Therapeutic opportunities for natural products in AD

Natural products are gaining increasing attention in AD therapy since there is almost no effective cure to date [104], and more than 100 natural compounds have been proposed as promising candidates for alleviating AD [105]. Indeed, approximately 46% new drugs that approved by FDA between 1981 and 2014 were from natural products and their derivatives [106]. Five compounds with huge therapeutic potential in AD intervention through redox signaling-associated mechanisms are highlighted as follows.

Curcumin, a natural phenolic compound isolated from the rhizomes of *Curcuma longa L,* is the most widely studied natural product in numerous diseases including AD as evinced by almost 9000 citations in the literature [107]. Curcumin could slow AD progression by inhibiting Aβ production [108], preventing Aβ aggregation [109] as well as destabilizing Aβ fibrils [110] and promoting Aβ clearance [111]. Moreover, it ameliorated cognitive impairment via suppressing tau hyperphosphorylation [112], inflammation and oxidative stress [113] as well. Unfortunately, although curcumin showed promising preclinical results, it failed to improve cognitive function in clinical trials [114]. The low bioavailability of curcumin and poor design of these trials may be speculated as factors that significantly limit its effect in humans, while nanoformulations [115] and exosomes [112] that enhance curcumin bioavailability might improve clinical outcomes. Future studies that aim to clarificate whether poor clinical benefit is due to its undesirable bioavailability or the inefficacy of curcumin on AD are desperately needed.

Quercetin [116], resveratrol [117] and ferulic acid [118] are other polyphenolic compounds isolated from vegetables and fruits with beneficial properties for AD treatment via inhibiting Aβ production/aggregation, destabilizing Aβ fibrils, alleviating oxidative stress and inflammation. Quercetin [119] and resveratrol [120] also rescued cognitive deficits by suppressing tau phosphorylation. In addition, resveratrol has been safe and well-tolerated for mild to moderate AD patients in two randomized, double-blind, placebo-controlled trials with uncertain efficacy on clinical outcomes [121, 122], while quercetin and ferulic acid are not studied in clinical trials so far.

Huperzine A, a natural product isolated from *Huperzia serrata* (Qian Ceng Ta), has been widely used for the inhibition of numerous diseases in China for centuries, which could attenuate or reverse cognitive deficits by selectively increasing acetylcholine levels as a potent and specific acetylcholinesterase inhibitor in a wide range of animal models [123]. In addition to the suppression of acetylcholinesterase, huperzine A also improved cognitive function via inhibiting Aβ production, ameliorating tau hyperphosphorylation [124], reducing inflammation and brain iron accumulation that further led to oxidative stress [125]. The phase IV clinical trials made in China demonstrated that Huperzine A (200 μg BID, 8w) significantly ameliorated cognitive dysfuntion in AD patients with slight peripheral cholinergic side effects [126, 127], while Rafii et al. discovered that huperzine A (200 μg BID, 16w) was ineffective in AD treatment [128], suggesting large-scale, randomized and placebo-controlled trials are required to further assess the effects of huperzine A on AD. Moreover, given the side effect of huperzine A on peripheral nerve system, N-[2-hydroxy-3-methoxy-5-chlorobenzylidene] huperzine A, a prodrug of huperzine A with minimal peripheral cholinergic effects, has been screened through a series of structural modification, which may facilitate the clinical use of huperzine A.

### The benefit of AD intervention on its complications

AD alleviation could be instrumental to other diseases as well since AD is a widespread systemic disease that could affect peripheral tissues or organs beyond the brain [129]. Here, we only briefly expatiate heart failure and chronic kidney disease (CKD) that pose an enormous challenge to human health for the sake of brevity.

Heart failure is most responsible for hospital admission in older adults, which remarkably aggravates national financial burden [130]. By 2030, above 8 million people (1 in every 33) will project to heart failure and the total costs of heart failure are forecasted to increase from $31 billion to $70 billion between 2012 and 2030 in the United States [130]. Cognitive decline is highly prevalent in heart failure performed in 1152 Australian participants, affecting 54% patients with heart failure, and has a profound impact on poorer outcomes [131]. The 30-day readmission rate of heart failure patients accompanied by cognitive damage was significantly elevated than that of participants with heart failure without cognitive damage (26.8% vs 12.8%), highlighting the importance of cognitive function intervention in improving adverse prognosis [132].

AD is closely associated with CKD progression as well. CKD has been proposed as a significant and independent risk factor for the development of cognitive impairment in a meta-analysis of 54,779 participants [133], while cognitive deficits can be significantly improved or even reversed after renal function restoration [134]. Particularly, the prevalence of cognitive damage can be up to 87% in end-stage renal disease patients [135]. The kidney and brain share common risk factors for microvascular damage since they have similar microvascular structure and hemodynamic fluctuation, including inflammation and oxidative stress [136], which might be helpful in understanding why CKD patients are inclined to develop neurological disorders. Therefore, it is intensely recommeded that busy nephrologists spend time in assessing cognitive function. Indeed, given the available treatments for AD are of limited efficacy, the high burden of cognitive dysfunction may partly explain why clinical performance targets of CKD have been so hard to achieve. A series of natural products isolated from diuretic traditional Chinese medicines in our previous studies, such as *Alisma orientale*, *Poria cocos* and *Semen Plantaginis*, showed promising efficacy in halting CKD [137–141]. Given the paramount association between CKD and AD, they may be beneficial to the recovery of AD as well.

## Concluding remarks

AD is the most common multifarious neurodegenerative disease and patients suffering from AD show a gradual loss of memory due to neuronal impairment. Inflammation and oxidative stress are major risk factors of AD, which play a particularly paramount role in AD development. In this review, we mainly focused on how inflammation and oxidative stress were involved in the pathogenesis of AD and the current knowledge concerning the potential association between redox signaling and metabolic pathways, providing additional evidence for biomarker identification and pathomechanism exploration. Moreover, considering the fact that there is almost no disease-modifying therapies for AD and the prominent role of inflammation and oxidative stress in cognitive dysfunction, we also highlighted the main redox signaling-associated mechanisms and their cross-talk in AD progression, aiding the exploitation of mechanism-based therapies.

Natural products are precious treasure for new drug discovery, which should be back into the spotlight again as the side effect of available commercial medicines often brought risks. Curcumin, Quercetin, resveratrol, ferulic acid and huperzine A showed promising future on AD through redox signaling-associated mechanisms, providing protential candidates for drug discovery. Undoubtedly, natural products are fertile ground for the development of pharmaceutical exploitation, while the paucity of randomized, placebo-controlled and well-designed trials in human severely restrict their clinical use. Considering natural products cannot be used in human until enough clinical data, there is intense impetus to combine preclinical evidence with clinical trails. Additionally, it is intensely recommeded that busy nephrologists and cardiologists spend time in assessing cognitive function since AD is highly prevalent in CKD and heart failure, the alleviation of which may facilitate CKD/heart failure recovery and improve clinical outcomes before diseases become advanced.

## Data Availability

Not applicable.

## Abbreviations

Aβ: β-amyloid
AD: Alzheimer’s disease
APOE: Apolipoprotein E
CKD: Chronic kidney disease
CSF: Cerebrospinal fluid
DAO: D-amino acid oxidase
NF-κB: Nuclear factor-κB
NMDAR: N-methyl-D-aspartate receptor
NOX: Nicotinamide adenine dinucleotide phosphate oxidase
Nrf2: Nuclear factor-erythroid 2 related factor 2
ROS: Reactive oxygen species
TGF-β: Transforming growth factor-β
